# Report of Three Cases of Amaurosis Produced by Tobacco

**Published:** 1863-11

**Authors:** J. C. Wordsworth

**Affiliations:** Surgeon to the Royal London Ophthalmic Society; Queen Anne Street


### REPORT OF THREE CASES OF AMAUROSIS PRODUCED BY TOBACCO.

By J. C. WORDSWORTH, Esq., F.R.C.S., Surgeon to the Royal London Ophthalmic Society.

Reprinted from the "London Lancet."

Case 1.—W. A——, aged 21, a clerk, residing at Liverpool, came to the Royal London Ophthalmic Hospital in 1861, on account of partial loss of sight in both eyes. He is a strong, healthy-looking, rather little man. Has always had excellent health, and never suffered from syphilis. His employment is principally in the open air, as he is engaged in clearing vessels at the Custom House, &c. For some years he has smoked, having gradually increased from two or three pipes per day, until he has reached the enormous amount of a pound to a pound and a-half of strong tobacco in the week; and for some time has rarely been without his pipe half an hour in the day. For a long period his sight has gradually failed, till he can only see to read, for a short time, characters of one-third of an inch. Though he has had misgivings that his ailment proceeded from tobacco-smoking, he has continued the habit to the present time, and is now daily becoming more blind.

Both pupils are rather large, but the motions of the irides are active. By means of the ophthalmoscope, both optic nerves appear of brilliant white color, their areas being enlarged, and their outlines irregularly defined.

Case 2.—J. M——, aged 36, a railway servant, came to the Ophthalmic Hospital, on account of dimness of sight in both eyes, about June, 1862. He is a tall, muscular, rather pale man, and says he has always had good health. He is employed as a signal-man, and has been accustomed to beguile his time by smoking all day long. For an uncertain time he has noticed his sight to be gradually failing, and attributed the defect to the use of tobacco. He has still continued to smoke to the present time, and his sight has now become so imperfect that he is unable to attend to his business. He has never had venereal disease of any kind, nor has he used his eyes much for close vision.

The pupils are considerably dilated, and not much influenced by light. The fundus of each eye seems quite normal, with the exception of the optic discs, which appear too large, and irregularly circular, the tissue being quite of tendinous whiteness.

Case 3.—G. A——, aged 28, a butcher, residing in Essex, applied at the Royal London Ophthalmic Hospital, March 25th, 1863, on account of failing sight in both eyes. He is a stout, strong, middle-sized man, having every appearance of health, and says he has had excellent health all his life. He began to smoke about eight or nine years ago, moderately, but, gradually increasing, has now for some time been in the habit of smoking half an ounce of strong tobacco every day, apparently without any ill effect. About nine months since, his sight began gradually to fail, and has continued to get worse to the present time. He has always been temperate as to the quantity of beer, &c., which he has taken, and has never drunk spirit habitually. He is a married man, and has three healthy children. Has never suffered from syphilis, nor has he used his eyes much at any trying occupation. With the exception of both pupils being rather large, and the motions of the irides sluggish, he has no external appearance of any ailment of the eyes. He can only see to read No. 18 test-type (canon) with his left eye, and with the right No. 16 (two-line great primer), word by word; and distant objects are equally indistinct.

The ophthalmoscope demonstrates an atrophic condition of both optic nerves, the inner (apparent) half of each, seen in the reversed image, being quite white and non-vascular; the outer part being redder, and more vascular than normal.

Within the last three years I have seen a considerable number of cases of amaurosis, apparently produced by the influence of tobacco. I admit (I need scarcely say) how difficult it is to reduce the etiology of this obscure affection to a demonstration. For, in the first place, amaurosis is attributed to a vast variety of causes, many of which are always more or less in operation; then, again, the disease is dependent on a similar variety of *pathological conditions;* and, lastly, our knowledge of the physiology as well as of the pathology of the retina and brain is so limited that we can ill appreciate or define the influence of physiological agents on their structures and functions.

No one can doubt that tobacco possesses properties that are capable of producing great effects on the nervous system at large, nor that the habitual use of it has much influence, of an indirect nature, on the vital reactions. Our only wonder is, that the almost universal employment of this powerful agent does not leave vestiges of its influence that are better known and recognized as signs of disease. This may be accounted for to some extent by the rapid cadaveric changes that occur in the nervous elements, thus obscuring or effacing diseased states before we have the opportunity of recognizing them.

All the classic writers attribute its full share of causation to tobacco as a source of amaurosis; yet I have not met many that are willing, individually, to allow that they have traced its influence. But it has often happened that the causes of disease are long unrecognized by many, after as full a proof has been made of their reality as possible. For instance, it is recorded of one of the causes of iritis (that every one now allows) that for many years it was not admitted by men of vast experience that any closer relation than that of coincidence existed between it and syphilis; yet so great has been the revulsion of opinion that some eminent men now seem to think it never occurs except in connexion with that contamination.

I have selected the cases above sketched to illustrate this subject, because they seem to be as free from the unavoidable fallacies that encircle this subject as possible. Many have come under my notice in which I could not find any other cause to account for the conditions; but few so typical of the atrophy of the optic nerve, or so advanced. It is obviously desirable to cite well-marked cases. Many of those observed gradually merged into less definite conditions, and were only corroborative, rather than conclusive. Again, many were so fettered with other complications that I consider them inapposite for my present purpose. All the cases that have come under my observation have (as might probably be expected) been in males. It will be noticed that only one pathological condition was seen in these three cases,—namely, that of white atrophy of the optic nerves. I am not prepared to assert that tobacco produces blindness in this way only; but in all my cases I have recognized this condition in a great or small degree.

I may anticipate that I shall be asked, How can it be that of the hundreds of thousands of smokers, only so small a proportion are affected by amaurosis? I should reply, first, that few probably smoke to such excess the strongest tobacco; in the second place, we are not yet in a position to recognize the smaller degrees of tobacco-disease; and, thirdly, as Dr. Mackenzie has aptly observed, only one of five hundred shall become amaurotic, in whom a stronger predisposition to the disease had existed.

Secondary syphilis affects the retina, and leads to amaurosis; but of the thousands affected how few become blind!

Then it has been suggested that I ought to show that amaurosis is most common where smoking is most general. To this I reply, it is impossible so to estimate and proportion the other recognized causes of amaurosis so as to enable us to compare them with the effects of tobacco, and thence deduce any relation. But so far as probability warrants, I think there may be some conclusion to this purpose deduced from the greater frequency of atrophy of the optic nerves in men than in women, (of which I suspect there is little doubt,) though the other causes of amaurosis are more likely to affect the latter,—for instance, needlework, &c.

Dr. Mackenzie, in his great work on Ophthalmology, expresses his belief that tobacco is a *frequent* cause of amaurosis, and adds that "one of the best proofs of tobacco being a cause of amaurosis is in the great improvement in vision—sometimes complete restoration—which ensues on giving up the use of this poison," and cites a very striking case in illustration. With him I agree also in the conviction, that tobacco is a common cause of the cases of partial loss of sight that are daily to be found at our hospitals.

Queen Anne Street, 1863.

